# Automating Clavien–Dindo classification with large language models in percutaneous nephrolithotomy: an exploratory study

**DOI:** 10.1007/s00345-026-06833-z

**Published:** 2026-09-28

**Authors:** Gustavo Perrone, Alexandre Danilovic, Carlos Batagello, Fabio Cesar Miranda Torricelli, Fabio Carvalho Vicentini, Giovanni Scala Marchini, Rodrigo Perrella, William C. Nahas, Eduardo Mazzucchi

**Affiliations:** 1 Faculdade de Medicina FMUSP, Universidade de Sao Paulo, Sao Paulo, SP Brazil; 2 Hospital das Clinicas HCFMUSP, Faculdade de Medicina, Universidade de Sao Paulo, Av. Dr. Eneas de Carvalho Aguiar, 255, sala 7175, Sao Paulo, SP 05403-000 Brazil

**Keywords:** Artificial intelligence, Large language models, Percutaneous nephrolithotomy, Postoperative complications

## Abstract

**Purpose:**

To explore the use of large language models for Clavien-Dindo classification of complications following percutaneous nephrolithotomy by benchmarking their performance against physician grading.

**Methods:**

We evaluated ChatGPT, Copilot, DeepSeek, Gemini, Grok and Llama for defining Clavien-Dindo grades, classifying complications according to the Clavien-Dindo classification, and classifying case summaries with and without in-context prompting. Vignettes were obtained from a published consensus categorization of percutaneous nephrolithotomy complications. We measured agreement with Gwet’s AC2 coefficient and compared it with attending urologists and urology residents.

**Results:**

All models accurately defined the Clavien-Dindo classification and graded with almost perfect agreement complication descriptions and case summaries (0.97 to 0.98 overall agreement). Six attendings and 15 residents classified the same vignettes, demonstrating lower agreement than the chatbots (0.95, *p* < 0.001 for residents; 0.96, *p* = 0.03 for attendings; 0.95, *p* < 0.001 for all participants). Models’ latency ranged from 0.77 to 3.90 min, while the fastest physician required 17 min to complete the same tasks. Models’ intrarater reliability was almost perfect: from 0.97 to 0.99. Interrater reliability between chatbots (0.97, 95% confidence interval: 0.97–0.98) was significantly higher than among physicians (0.93, 95% confidence interval: 0.92–0.94).

**Conclusion:**

Large language models show promise in being faster, more accurate, and precise than physicians in Clavien-Dindo classification in an experimental setting. We support the need for a prospective agreement study using real-world clinical records. We urge discussion of the legal and ethical implications.

**Supplementary Information:**

The online version contains supplementary material available at https://doi.org/10.1007/s00345-026-06833-z.

## Introduction

The Clavien-Dindo classification is highly recommended in surgical studies. This classification has become a universal language for reporting postoperative complications [1]. More importantly, it serves as a common denominator that enables comparisons across different procedures, surgical techniques, or even postoperative outcomes in different patient populations.

However, there is a critical limitation: despite numerous published efforts to establish consensus, interrater reliability remains only fair to moderate in Urology [2, 3] and subfields such as Prostate Surgery [4], Endourology [5] and Pediatric Urology [6]. This raises questions about the reliability of any conclusions that use Clavien-Dindo grading as a study endpoint.

We aimed, as a feasibility study, to explore the use of large language models (LLMs, or chatbots) for Clavien-Dindo classification of complications following percutaneous nephrolithotomy (PCNL) and benchmark their performance against physician grading. We hypothesize that LLMs are faster, more accurate and precise than physicians at classifying postoperative complications using the Clavien-Dindo system. Thus, they may offer a promising alternative to overcome the lack of agreement in surgical outcome reporting.

## Materials and methods

### Study materials and population

In December 2025, we tested ChatGPT-5.2 (OpenAI, San Francisco, CA, United States), Copilot (Microsoft Corporation, Redmond, WA, United States), Gemini 3 (Google LLC, Mountain View, CA, United States), Grok 4.1 (xAI Corp., Palo Alto, CA, United States), DeepSeek V3.2 (Hangzhou DeepSeek AI Technology Co. Ltd., Hangzhou, ZJ, China), and Llama 4 (Meta Platforms Inc., Menlo Park, CA, United States). The first four were proprietary (closed-source) models, whereas the latter two were open-weight models. We used the free, standard (non-reasoning), publicly available versions of these models without any additional fine-tuning.

Following ethical approval by the institutional review board (91048325.0.0000.0068), these chatbots were compared with attending urologists from the Endourology Unit, which performs over 250 percutaneous procedures annually, and Urology residents at our referral center. Participants (hereafter referred to as physicians) were invited via e-mail in September 2025 to voluntarily complete an anonymous electronic survey (SurveyMonkey Inc., San Mateo, CA, United States) after providing informed consent.

Clavien-Dindo grades followed the 2004 revision of the 1992 system [7, 8]. We added grade “0” for no complications, distinguished IIIa/IIIb and IVa/IVb, and disregarded the “d” suffix for disability at discharge, totaling eight categories. All data concerned postoperative complications following PCNL.

### Primary outcome: LLMs’ reasoning agreement

First step. We asked the chatbots to define Clavien-Dindo grades. This step was not applied to physicians; instead, they were provided with the grade definitions before the survey.

Second step. We asked chatbots and physicians to classify 68 postoperative complications from a Clavien-Dindo categorization on PCNL [5]. We then compared the responses of each chatbot and physician to the mentioned categorization and calculated a match ratio. Notice that misclassifying a grade I as grade II is quite different from misclassifying it as grade IVa. Beyond simple correctness, we considered the degree of deviation using Gwet’s AC2 coefficient with ordinal weights [9, 10]. We classified strength of agreement according to Landis and Koch [11].

Third step. We also compared chatbot answers with our own consensus, that is, attendings’ modal response for each vignette. Ties were resolved using residents’ modal response. We used for this step 69 clinical case summaries published alongside the mentioned Clavien-Dindo consensus for PCNL [5]. The original article included 70 cases; however, we excluded the case describing a patient with an “unknown complication”, resulting in 69 cases for our analysis. We applied the same statistical analyses as described above.

Fourth step. Lastly, we investigated whether agreement could be improved via in-context prompting. Each chatbot was provided with a reference list of complications (the same presented in the *Second step*) and corresponding Clavien-Dindo grades. We then presented the same case summaries shown in the *Third step* and asked the chatbot to classify them based on this reference. We applied the same procedure to the survey. Improvement was tested using a bootstrap paired t-test [12], comparing AC2 coefficients from the *Third* and *Fourth step*.

Comparison of LLMs versus physicians. A single AC2 coefficient, encompassing *Second* and *Third step*, was used to measure overall agreement and compare LLMs with physicians by a generalized parametric bootstrap test [13], with rater category (LLM, attending, or resident) as a fixed effect. The *Fourth step* was excluded from the overall coefficient to avoid double counting the same vignettes used in the *Third step* and was instead presented as a sensitivity analysis. *Post hoc* pairwise comparisons were adjusted using Holm correction. We documented attendings’ years in practice and tested the correlation with overall agreement using a bootstrapped Pearson’s correlation [14].

Models were accessed through their publicly available web-based interfaces in Safari private browsing. Web-browsing was disabled. New chatbot accounts were created to ensure memory-free environments. Each prompt, developed de novo by the authors for this study, was submitted in a new chat window, with the context reset between each step. We used the same prompts across all chatbots and for the physicians’ survey to ensure consistency (prompts are provided as supplementary material). A single researcher conducted all interactions to maintain uniformity. Vignettes were shuffled to prevent chatbots and participants from inferring Clavien-Dindo grades based on the order of presentation and followed the same order for both physicians and chatbots. In the survey, backward navigation between steps was disabled, and all items were mandatory. All statistical analyses were conducted in R 4.5.0 [15], using two-tailed tests, with a significance level of 5% and 95% confidence intervals (CIs). For bootstrap tests, 10^5^ iterations were performed.

### Secondary outcomes: LLMs’ reasoning capacities

We also evaluated LLMs based on their reasoning capacities:

Disclaimers. We defined as whether the chatbot issues appropriate warnings about sharing patient data or reminds users that classifications should be made by qualified healthcare professionals.

Artificial intelligence (AI) hallucinations. We defined as instances where the chatbot fabricated facts or provided responses that were unrelated to the prompts.

Latency. We defined as the time taken by the chatbot to respond to the given command. It was calculated as the sum of the time required for *Second* to *Fourth step*. For physicians, the survey captured the overall completion time (from the *Second* to *Fourth step*), excluding time spent reviewing the Clavien-Dindo grade definitions.

Persuadability. We asked the chatbots, for each wrong answer in the *Second step*, if the complication could be classified as the correct grade. We defined *persuadability* as whether the chatbot can be persuaded to revise an incorrect answer, or if it persists in its initial (wrong) response. We presented it as the ratio of wrong answers that were persuaded to the total number of wrong answers.

Reliability. We repeated all prompts in fresh, reset sessions. We calculated the match ratio and AC2 coefficient between the first and second runs. We evaluated interrater reliability among different LLMs measuring agreement across the first run of all chatbots. For physicians, interrater reliability was calculated across residents, attendings, and all participants. Differences between physicians’ and LLMs’ interrater reliability were considered statistically significant if their 95% CIs did not overlap.

Weighability. We defined as the chatbot’s ability to spontaneously consider alternative classifications. We presented it as the ratio of the number of weighable classifications over the total number of classifications.

## Results

### LLMs’ reasoning agreement

First step. All chatbots accurately defined the Clavien-Dindo grades. All except Llama described the distinctions between grades IIIa/IIIb and IVa/IVb, although Llama recognized these distinctions when specifically prompted. ChatGPT and Llama did not initially mention the “d” suffix indicating disability at discharge but provided correct explanations when asked. None of the chatbots proactively addressed how to classify multiple complications; however, all responded correctly when prompted. Notably, Copilot, Gemini, and Grok suggested using the Comprehensive Complication Index in such cases, despite evidence showing limited benefit in PCNL studies [16].

**Fig. 1:**
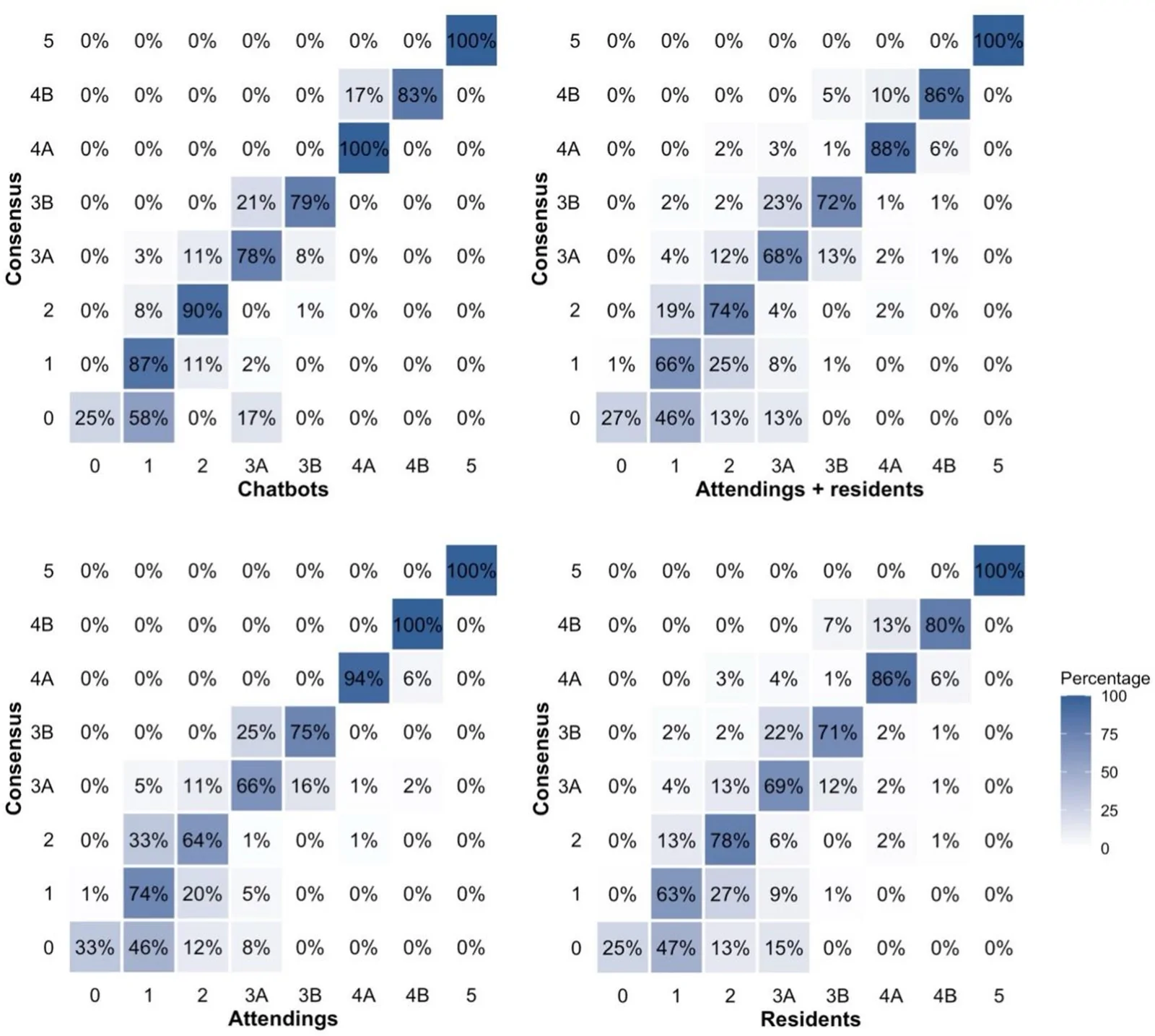
Confusion matrices comparing chatbots’, attendings’ and residents’ classifications in the *Second step* with the consensus. A blue-shaded heatmap was used to enhance visualization. Chatbots showed greater difficulty differentiating grades IIIa and IIIb. Attending urologists and residents also showed lower agreement for IIIa versus IIIb but additionally showed greater difficulty distinguishing grades I and II. Across all groups, the lowest agreement was observed for grade 0 (no complication)

Second step. All chatbots classified the complication descriptions with almost perfect agreement with the reference standard, defined as AC2 > 0.80 [11]. Table 1 presents the match ratios and agreement coefficients per chatbot. Chatbots misclassified minor complications (0–II) as major (IIIa–V) in 6.0% of cases, and major complications as minor in 5.5%. Figure 1 shows chatbots’ classifications confusion matrix in the *Second step* against the consensus.

Third step. Chatbots also performed with almost perfect agreement (AC2 > 0.80) relative to our own consensus (attendings’ modal response for each vignette, with residents’ modal response used to break ties in 9/69 case summaries).

Fourth step. After being presented with a list of categorized complications for in-context prompting, all chatbots maintained an almost perfect classification of the case summaries (AC2 > 0.80). We do not have evidence of improvement in agreement when comparing zero-shot with in-context prompting (*p* = 0.11).

**Table 1:** Agreement by large language model. AC2 coefficient with 95% confidence intervals. Match ratios are reported for descriptive purposes only; as explained in the *Materials and Methods* section, agreement should be assessed using AC2 coefficients. Misclassifications include the *Second* and *Third steps*

		Classifying 68 complication descriptions (*Second step*)	Classifying 69 case summaries		Overall agreement (137 vignettes)	Misclassifications	
			Zero-shot prompting (*Third step*)	In-context prompting (*Fourth step*)		Minor (0-II) as major (IIIa-V)	Major (IIIa-V) as minor (0-II)
ChatGPT	Match ratio	81% (55/68)	86% (59/69)	81% (56/69)	83% (114/137)	7.1% (5/70)	4.5% (3/67)
	AC2	0.95 [0.92, 0.99]	0.97 [0.96, 0.99]	0.97 [0.95, 0.99]	0.97 [0.95, 0.99]		
Copilot	Match ratio	87% (59/68)	77% (53/69)	80% (55/69)	82% (112/137)	8.6% (6/70)	4.5% (3/67)
	AC2	0.98 [0.97, 0.99]	0.94 [0.92, 0.97]	0.96 [0.94, 0.99]	0.97 [0.96, 0.98]		
DeepSeek	Match ratio	76% (52/68)	81% (56/69)	83% (57/69)	79% (108/137)	2.9% (2/70)	6.0% (4/67)
	AC2	0.96 [0.93, 0.98]	0.98 [0.97, 0.99]	0.98 [0.97, 0.99]	0.97 [0.96, 0.98]		
Gemini	Match ratio	82% (56/68)	86% (59/69)	87% (60/69)	84% (115/137)	0% (0/70)	7.5% (5/67)
	AC2	0.97 [0.95, 0.99]	0.97 [0.96, 0.99]	0.99 [0.98, 1.00]	0.98 [0.97, 0.99]		
Grok	Match ratio	85% (58/68)	81% (56/69)	81% (56/69)	83% (114/137)	10.0% (7/70)	3.0% (2/67)
	AC2	0.97 [0.94, 0.99]	0.97 [0.95, 0.99]	0.98 [0.97, 0.99]	0.97 [0.95, 0.98]		
Llama	Match ratio	82% (56/68)	81% (56/69)	81% (56/69)	82% (112/137)	7.1% (5/70)	7.5% (5/67)
	AC2	0.98 [0.97, 0.99]	0.95 [0.93, 0.98]	0.96 [0.94, 0.98]	0.97 [0.96, 0.98]		

### Physicians’ grading

Six out of eight (75%) attending urologists, with a median of 17 years in practice (interquartile range 14 to 20), and all 15 (100%) Urology residents, with 5 residents from each year of training, completed the survey. All participants classified complication descriptions and case summaries with almost perfect agreement with the reference standard (AC2 > 0.80). See Fig. 1 for confusion matrices of attendings, residents, and all participants. There is no evidence of physician’s improvement after viewing the consensus list of complications (*p* = 0.7 for residents, *p* = 0.2 for attendings, *p* > 0.9 for all participants). We did not find a correlation between agreement with the reference standard and years in practice (*p* = 0.7). Table 2 describes their performance.

**Table 2:** Physicians’ grading. Data are shown as median (first, third quartile), except interrater reliability, shown as AC2 coefficient [95% confidence interval]. Match ratios are reported for descriptive purposes only; as explained in the *Materials and Methods* section, agreement should be assessed using AC2 coefficients. Misclassifications include the *Second* and *Third steps*

*n*			Urology residents	Attending urologists	All (residents and attendings)
			15	6	21
Classifying 68 complication descriptions (*Second step*)		Match ratio	74% (63, 76)	70% (69, 72)	72% (69, 76)
		AC2	0.94 (0.94, 0.95)	0.95 (0.94, 0.96)	0.94 (0.94, 0.96)
Classifying 69 case summaries	Before viewing consensus (*Third step*)	Match ratio	70% (64, 76)	71% (67, 74)	70% (65, 75)
		AC2	0.95 (0.92, 0.96)	0.95 (0.93, 0.96)	0.95 (0.92, 0.96)
	After viewing consensus (*Fourth step*)	Match ratio	77% (68, 80)	78% (64, 79)	77% (67, 80)
		AC2	0.96 (0.93, 0.97)	0.97 (0.94, 0.97)	0.96 (0.93, 0.97)
Overall agreement (137 vignettes)		Match ratio	71% (65, 75)	70% (69, 74)	71% (66, 74)
		AC2	0.95 (0.93, 0.96)	0.96 (0.94, 0.96)	0.95 (0.93, 0.96)
Misclassified minor (0-II) as major (IIIa-V)			9.8% (103/1,050)	6.4% (27/420)	8.8% (130/1,470)
Misclassified major (IIIa-V) as minor (0-II)			8.6% (86/1,005)	6.2% (25/402)	7.9% (111/1,407)
Interrater reliability (AC2)			0.93 [0.91, 0.94]	0.94 [0.92, 0.95]	0.93 [0.92, 0.94]
Time (minutes)			29 (23, 34)	39 (35, 66)	30 (23, 43)

Comparison of LLMs versus physicians. LLMs demonstrated higher agreement with the reference standard than residents (*p* < 0.001), attendings (*p* = 0.03) and residents and attendings combined (*p* < 0.001). There is no evidence of difference between attendings and residents (*p* = 0.3). As a sensitivity analysis, defining overall agreement across the *Second* to *Fourth steps*, LLMs showed higher agreement than residents and attendings combined (*p* < 0.001) and residents alone (*p* = 0.006), but not attendings (*p* = 0.13). In a second sensitivity analysis defining overall agreement across the *Second* and *Third steps*, while excluding nine *Third step* items that required tie-breaking by residents, LLMs again showed higher agreement than residents (*p* = 0.002) and residents and attendings combined (*p* < 0.001), but not attendings (*p* = 0.051).

### LLMs’ reasoning capacities

Disclaimers. When asked to classify complications, Copilot disclaimed “this is a general educational classification, not personalized medical advice”. The remaining chatbots provided answers without disclaimers.

Hallucinations. None of the chatbots fabricated facts or provided responses that were unrelated to the prompts.

Latency. All chatbots exhibited a latency under four minutes. The fastest physician, a resident, required 17 min to complete the same tasks.

Persuadability. Initially, Grok classified bladder catheterization due to urine retention as grade IIIa. When actively asked if it could be grade I, it revised its answer, as “catheterization is a simple bedside procedure”. This response reflects what we call *persuadability*.

Reliability. Intrarater reliability was almost perfect across all chatbots. Interrater reliability between LLMs was 0.97 (95% CI: 0.97–0.98), significantly higher than that observed among physicians (0.93, 95% CI: 0.92–0.94). Interrater reliability was higher among attendings than among residents, although confidence intervals overlapped.

Weighability. When prompted to classify a perirenal abscess drainage, Copilot responded that it could be grade IIIa if percutaneous, or IIIb if an open drainage was performed. This response illustrates what we define as *weighability*. Table 3 summarizes all reasoning dimensions evaluated for each LLM.

**Table 3:** Reasoning capacity dimensions by large language model. AC2 coefficient with 95% confidence intervals. Note that intrarater reliability encompasses *Second* and *Third steps* (137 vignettes), whereas weighability encompasses the *Second*, *Third*, and *Fourth steps* (206 vignettes)

Dimension	ChatGPT	Copilot	DeepSeek	Gemini	Grok	Llama
Disclaimers	No	Yes	No	No	No	No
Hallucinations	No	No	No	No	No	No
Latency (minutes)	1.08	1.59	3.90	1.30	0.77	1.57
Persuadability	77% (10/13)	33% (3/9)	12% (2/16)	58% (7/12)	30% (3/10)	50% (6/12)
Intrarater reliability	88% (121/137), AC2 = 0.98 [0.96, 0.99]	91% (125/137), AC2 = 0.99 [0.98, 1.00]	88% (121/137), AC2 = 0.98 [0.97, 0.99]	89% (122/137), AC2 = 0.99 [0.98, 0.99]	96% (131/137), AC2 = 0.99 [0.98, 1.00]	90% (123/137), AC2 = 0.97 [0.96, 0.99]
Weighability	0/206	0.5% (1/206)	1.0% (2/206)	2.4% (5/206)	0/206	0/206

## Discussion

LLMs demonstrate higher agreement than physicians in grading postoperative complications according to the Clavien-Dindo classification. Chatbots exhibit intrinsic knowledge of Clavien-Dindo grades and their application, obviating the need for additional definitions or explanations. All LLMs achieved almost perfect agreement when classifying not only complication descriptions but also case summaries.

Caution is warranted when interpreting comparative performance. While Copilot outperformed other chatbots in grading complication descriptions, and Gemini performed particularly well when using in-context prompting, these results do not imply they are inherently superior. It is important to avoid conclusions based solely on point estimates such as match ratios or the AC2 coefficient, as our results reflect only the performance in our dataset. To properly infer rankings, confidence intervals must be considered. Notice these intervals overlapped between models; we therefore cannot assert a hierarchy.

LLMs demonstrate greater precision than physicians in applying the Clavien-Dindo classification, not only when the same model is used (intrarater reliability) but also across different models (interrater reliability). Precision is a core requirement of any measurement method, as the Clavien-Dindo classification is for postoperative complications. In studies involving multiple investigators in data collection and, consequently, in grading complications, each study arm may be handled by different researchers. Multicenter studies encounter a similar problem. The complexity increases further when outcomes from several studies are reviewed, or when these data are synthesized in a meta-analysis. The possibility to employ a single, fatigue-resistant evaluator across all study arms, centers, and even across different studies represents an advancement in the standardization of outcome assessment. LLMs provide such a solution.

In the literature, human interrater reliability is reported to be 0.4 to 0.45 (Fleiss’ kappa) in General Urology [2, 3], 0.49 (Krippendorff alpha) in Pediatric Urology [6], 0.46 (Fleiss’ kappa) for PCNL [5] and 0.36 (Fleiss’ kappa) in prostate vaporization [4]. Outside Urology, interrater reliability tends to be higher: 0.64 to 0.88 (not reported) in Head and Neck Surgery [17, 18], 0.91 (Kendall’s W) in Pediatric Surgery [19] and 0.92 (not reported) in Bariatric Surgery [20]. These differences may partially reflect the use of unweighted rather than weighted agreement coefficients—a choice often unspecified in published studies. For example, we observed a physicians’ interrater reliability of 0.93 using AC2 with ordinal weights, which decreased to 0.62 when applying the unweighted AC1. Ultimately, it is conceptually inconsistent to validate the Clavien-Dindo classification employing an unweighted agreement coefficient and disregarding its ordinal nature [21].

Although the superiority of LLMs over physicians was statistically significant in both agreement and precision, this difference does not necessarily have meaningful practical implications, as both LLMs and physicians achieved almost perfect agreement for both metrics. Nevertheless, LLMs are not only effective, but also efficient: the marked reduction in classification time alone constitutes a compelling advantage for their use. The slowest chatbot was four times faster than the fastest physician. Admittedly, LLM latency does not account for operator time and therefore underestimates the total time required.

Our findings are consistent with those of Staubli and colleagues, showing slight improvements in agreement, latency and intrarater reliability [22]. These improvements may reflect advances in ChatGPT-5.2 (used by us) compared to ChatGPT-4 (used by them). Unlike their study, we did not observe any AI hallucinations, which may indicate further improvement in the newer version. The shorter latency observed in our study may be partially explained by our use of two-sentence summaries, rather than full case vignettes as in their methodology. Notably, they reported 0.92 agreement between human and ChatGPT classifications of real-world discharge summaries.

Hsueh and colleagues reported a lower match ratio when evaluating ChatGPT-4 [23]. This discrepancy may be explained by their use of real-world discharge summaries, a more complex task than assessing individual complications. A further limitation is that, while Staubli and our study relied on previously published data (consensus-based grading), Hsueh compared the chatbot’s output to human evaluators, without further specifying their qualifications. Nonetheless, both Staubli’s [22] and Hsueh’s [23] studies share the limitation of assessing only ChatGPT.

The major limitation to automating Clavien-Dindo classification with LLMs is the ethical concern of processing sensitive patient data on unsecured platforms. Hsueh and colleagues interestingly addressed this by developing an in-house Python script for automatic deidentification of medical records [23]. On this matter, Staubli and colleagues also emphasized the need to balance over- and under-anonymization: removing too much information risks losing clinically relevant data, while insufficient anonymization can compromise patient privacy [22]. The use of AI in medicine deserves and requires timely, evidence-based discussion [24–26]. Recent studies have demonstrated reasonable performance in urological knowledge [27–29].

This study has limitations. First, LLMs were evaluated using previously published vignettes, which may have led to overestimation of agreement if the models had been exposed to these vignettes during training. High interrater agreement between models may also be overestimated because of shared training data, particularly in the case of Copilot, which incorporates OpenAI models alongside Microsoft’s own technologies. Second, chatbots classified complications based on two-sentence case summaries (the same used to validate the Clavien-Dindo classification for PCNL) [5]. Their performance with full discharge summaries—particularly when multiple complications are present and only the most severe must be graded—remains unknown, as these could not be assessed due to ethical concerns. Agreement would likely decrease and response time increase, although human raters would likely experience similar effects. Third, all vignettes concerned complications of PCNL, so generalizability to other procedures or surgical fields is uncertain. However, extrapolation is reasonable, as many of the complications overlap with those seen in other urological and surgical procedures. Fourth, comparisons with attendings and residents from a single center introduce potential selection bias, besides potential nonresponse bias due to the two attendings who did not participate. Fifth, the attending group comprised only six physicians, limiting the robustness and generalizability of the findings. Sixth, all attending urologists were Endourology subspecialists with over a decade of experience. Comparisons with general urologists might reveal greater performance differences, although our findings with residents—representing the beginning of the training spectrum—suggest otherwise. Seventh, physicians’ intrarater reliability could not be assessed because the survey was administered only once to each participant. Eighth, using attendings’ modal response as the reference standard for the *Third* and *Fourth steps* introduces circularity and may inflate agreement for the attending group. Nevertheless, LLMs showed significantly higher agreement with the reference standard than physicians overall and outperformed both attendings and residents in pairwise comparisons. The attending-versus-resident comparison, which favored attendings, was not statistically significant. Importantly, this circularity affects only comparisons of agreement with the reference standard and does not affect reliability or other analyses.

## Conclusions

LLMs demonstrate potential for being faster, more accurate, and precise than physicians in Clavien-Dindo classification in an experimental setting. A major limitation is the potential overestimation of agreement due to prior exposure to these vignettes during training. As an exploratory study, with its discussed limitations, we cannot assert the performance of LLMs in real-world clinical practice. We advocate for a prospective agreement study using real-world clinical records. We urge discussion of the legal and ethical implications.

## Supplementary Information

Below is the link to the electronic supplementary material.

Supplementary Material 1

## Data Availability

Data and statistical code are available for bona fide researchers who request it from the authors.
