# Supplementary material for: Automating Clavien–Dindo classification with large language models in percutaneous nephrolithotomy: an exploratory study
Source: World J Urol. 2026 Sep 28;44(1):713. doi: 10.1007/s00345-026-06833-z (PMC13619659; doi:10.1007/s00345-026-06833-z)
Supplement: Supplementary file 1 [file 345_2026_6833_MOESM1_ESM.pdf]

SUPPLEMENTARY INFORMATION

Automating Clavien-Dindo Classification with Large Language Models: an exploratory study

World Journal of Urology

Gustavo Perrone[a], Alexandre Danilovic[b], Carlos Batagello[b], Fabio Cesar Miranda Torricelli[b], Fabio Carvalho Vicentini[b], Giovanni Scala Marchini[b], Rodrigo Perrella[b], William C Nahas[a], Eduardo Mazzucchi[b]

[a]Faculdade de Medicina FMUSP, Universidade de Sao Paulo, Sao Paulo, SP, BR

[b]Hospital das Clinicas HCFMUSP, Faculdade de Medicina, Universidade de Sao Paulo, Sao Paulo, SP, BR

Corresponding Author: Alexandre Danilovic, alexandre.danilovic@hc.fm.usp.br

Prompts

First step

Define the Clavien-Dindo classification

Second step

Act as an experienced endourologist engaged in scientific research.
I will provide you with a list of postoperative complications following percutaneous nephrolithotomy. Your task is to classify each complication using the Clavien-Dindo classification. For each complication, provide the corresponding Clavien-Dindo grade. Wait for me to provide the list before responding. Confirm your role and readiness

Third step

Act as an experienced endourologist engaged in scientific research.
I will provide you with a list of clinical case summaries of patients who underwent percutaneous nephrolithotomy. Your task is to classify each case using the Clavien-Dindo classification. For each complication, provide the corresponding Clavien-Dindo grade. Wait for me to provide the list before responding. Confirm your role and readiness

Fourth step

Act as an experienced endourologist engaged in scientific research.
I will first provide you with a reference list of postoperative complications following percutaneous nephrolithotomy (PCNL), already classified according to the Clavien-Dindo classification. Use this list as your learning reference and consensus standard.
After that, I will send you a list of clinical case summaries of patients who underwent PCNL. Your task is to classify each case using the Clavien-Dindo classification, based on the consensus reference I provided. For each complication, provide: the corresponding Clavien-Dindo grade. Wait for me to provide the list before responding. Confirm your role and readiness

CHART: Chatbot Assessment Reporting Tool

Reporting Checklist for Chatbot Health Advice Studies

Heading	Item #	Item	Page #
**Title & Abstract**			
Title	1a	State that the study is assessing one or more generative AI-driven chatbots for clinical evidence or health advice.	1
Abstract/Summary	1b	Apply a structured format, if applicable.	3-4
**Introduction**			
Background	2a	State the scientific background, rationale, and healthcare context for evaluating the generative AI-driven chatbot(s), referencing relevant literature when applicable.	5
	2b	State the aims and research questions including the target audience, intervention, comparator(s), and outcome(s).	5
**Methods**			
Model Identifiers	3a	State the name and version identifier(s) of the generative AI model(s) and chatbot(s) under evaluation, as well as their date of release or last update.	6
	3b	State whether the generative AI model(s) and chatbot(s) are open-source or closed-source/proprietary.	6
Model Details	4a	State whether the generative AI model was a base model or a novel base model, tuned model, or fine-tuned model.	6
	4b	If a base model is used, cite its development in sufficient detail to identify the model.	Not applicable
	4c	If a novel base model, tuned model, or fine-tuned model is used, describe the pre- and/or postimplementation/deployment data and parameters.	Not applicable

Prompt Engineering	**5a**	Describe the evolution of study prompt development.	Not reported
	**5ai**	Describe the sources of prompts.	8
	**5aii**	State the number and characteristics of the individual(s) involved in prompt engineering.	Not reported
	**5aiii**	Provide details of any patient and public involvement during prompt engineering.	Not applicable
	**5b**	Provide study prompts.	Supplementary material
Query Strategy	**6a**	State route of access to generative AI model.	8
	**6b**	State the date(s) and location(s) of queries for the generative AI-driven chatbot(s) including the day, month, and year as well as city and country.	6
	**6c**	Describe whether prompts were input into separate chat session(s).	8
	**6d**	Provide all generative AI-driven chatbot output/responses.	Not reported
Performance Evaluation	**7a**	Define the ground truth or reference standard used to define successful generative AI-driven chatbot performance.	7
	**7b**	Describe the process undertaken for generative AI-driven chatbot performance evaluation.	7-9
	**7bi**	State the number and characteristics of team members involved in performance evaluation.	Not reported
	**7bii**	Provide details of any patients and public involvement during the evaluation process.	Not applicable
	**7biii**	State whether evaluators were blinded to the identity of the generative AI-driven chatbot(s) under assessment.	Not reported
Sample Size	**8**	Report how the sample size was determined.	6

Data Analysis	**9a**	Describe statistical analysis methods, including any evaluation of reproducibility of generative AI-driven chatbot responses.	7-9
	**9ai**	Report the measures used for performance evaluation.	7-9
**Results**			
	**10a**	Report the performance evaluation undertaken including the alignment between generative AI-driven chatbot output and ground truth or reference standard using quantitative or mixed methods approaches as applicable.	10
	**10b**	For responses deviating from the ground truth or reference standard, state the nature of the difference(s).	Figure 1
	**10c**	Report the evaluation for potentially harmful, biased, or misleading responses.	15-16
**Discussion**			
	**11a**	Interpret study findings in the context of relevant evidence.	19-20
	**11b**	Describe the strengths and limitations of the study.	20-21
	**11c**	Describe the potential implications for practice, education, policy, regulation, and research.	18-19
**Open Science**			
Disclosures	**12a**	Report any relevant conflicts of interest for all authors.	23
Funding	**12b**	Report sources of funding and their role in the conduct and reporting of the study.	24
Ethics	**12c**	Describe the process undertaken for ethical approval.	23
	**12ci**	Describe the measures taken to safeguard data privacy of patient health information,	Not applicable

		as applicable.	
	**12cii**	State whether permission/licensing was obtained for the use of original, copyrighted data.	Not applicable
Protocol	**12d**	Provide a study protocol.	Not provided
Data availability	**12e**	State where study data, code repository, and model parameters can be accessed.	24
